# Regulatory features of *Candida albicans* hemin-induced filamentation

**DOI:** 10.1093/g3journal/jkae053

**Published:** 2024-03-12

**Authors:** Liping Xiong, Katharina Goerlich, Aaron P Mitchell

**Affiliations:** Department of Microbiology, University of Georgia, Athens, GA 30602, USA; Department of Microbiology, University of Georgia, Athens, GA 30602, USA; Department of Microbiology, University of Georgia, Athens, GA 30602, USA

**Keywords:** *Candida albicans*, filamentation, biofilm, gene regulation

## Abstract

*Candida albicans* is a prominent fungal pathogen that can infect the bloodstream and deep tissues. One key pathogenicity trait is the ability to transition between yeast and hyphal growth. Hyphae are critical for the formation of biofilms, which in turn enable device-associated infection. Among signals that drive hypha formation is the presence of hemin, an oxidized Fe(III)-containing heme derivative found in blood. In this study, we asked 4 questions. First, how uniform is the filamentation response to hemin among *C. albicans* strains? We tested 26 diverse isolates and found that the strength of a strain's filamentation response to hemin reflected its filamentation level in the absence of hemin. Second, does hemin induce biofilm formation? Hemin biofilm induction was evident in 5 out of 10 isolates tested, including most of the weaker biofilm formers tested. Third, what is the gene expression response to hemin? We compared RNA-seq data for type strain SC5314 grown in pH 5.5 minimal media with or without hemin. We also compared that response to SC5314 grown in pH 7.0 minimal media, where it undergoes well-studied pH-dependent filamentation. We found a common set of 72 genes with upregulated RNA levels in response to both signals, including many known hypha-associated genes. Surprisingly, overlap among those 72 genes with 2 recent consensus definitions of hypha-associated genes was limited to only 16 genes. Fourth, which regulators govern hemin-induced filamentation? A mutant survey indicated that the response depends upon filamentation regulators Efg1, Brg1, and Rim101, but not upon heme acquisition regulator Hap1 or its target genes *HMX1*, *RBT5*, *PGA10*, *PGA7*, and *CSA2*. These findings argue that hemin induces hypha formation independently of its utilization.

## Introduction

The ability to grow as hyphae is central to the pathogenicity of *Candida albicans* (Basso *et al*. 2019). Hypha formation is required for virulence in most animal infection models. Hyphae are also critical for formation of biofilms, the growth state that leads to device-associated infection (Desai *et al*. 2014). The impact of hyphae on virulence and biofilm formation is in part due to genes that are expressed at much higher levels in hyphae than yeast, called hypha-associated genes (Noble *et al*. 2017; Basso *et al*. 2019). The products of hypha-associated genes include surface adhesins (de Groot *et al*. 2013), the secreted toxin Candidalysin (Naglik *et al*. 2019), and secreted hydrolases (Jackson *et al*. 2007; Mayer *et al*. 2013) that can promote tissue destruction.

Hypha formation can be induced by diverse environmental signals. Among signals that may be found in host niches are serum, high CO_2_, *N*-acetylglucosamine, nutrient limitation, high temperature, host protein PTMA, surface contact, bacterial peptidoglycan, hypoxia, and neutral pH. These signals are connected to hyphal induction through a set of signaling pathways that govern expression or activity of multiple transcription factors. Most transcription factors that are required for hypha formation do not seem to be signal specific (Basso *et al*. 2019).

One host-relevant signal that induces hypha formation is hemin (Casanova *et al*. 1997), an oxidized Fe(III)-containing heme derivative that can be found in blood (Schaer *et al*. 2013). Hemin is a prospective iron source for *C. albicans*, with uptake mediated by a group of cell surface heme-binding proteins and a ferric reductase. The corresponding genes are activated by the transcription factor Hap1, as is the heme oxygenase gene *HMX1* (Andrawes *et al*. 2022). However, the genes that mediate hemin-induced hypha formation have not been investigated to our knowledge.

In this study, we characterized the induction of hypha formation by hemin. We examined the extent of strain variation in hemin-induced hyphal morphogenesis and biofilm formation. We also characterized the gene expression response to hemin through RNA-seq under growth conditions that enable hemin-induced filamentation. Finally, we examined a panel of mutants for the hemin-induced filamentation response.

## Methods

### Media and culture conditions

A full list of strains used in this study is in Supplementary Table 1. Strain stocks were maintained in 15% glycerol at −80°C. The media used in this study included YPD media (2% dextrose, 2% Bacto peptone, and 1% yeast extract), YPD + NAT [YPD supplemented with 400 μg/mL nourseothricin (clonNAT, Gold Biotechnology)], synthetic SD media (2% dextrose, 0.67% Difco yeast nitrogen base without amino acids and ammonium sulfate), and CSM media (2% dextrose, 0.67% Difco yeast nitrogen base without amino acids and ammonium sulfate, and 0.079% MP CSM). Media were solidified with 2% Bacto agar when necessary. Streaked strains were grown on YPD agar plates, and overnight cultures were grown in liquid YPD media at 30°C with shaking. Transformants were selected on synthetic SD media with any necessary auxotrophic supplements or on YPD + NAT agar plates.

Because hemin powder (Sigma-Aldrich H9039) is dissolved in 1.4 N NaOH to make a stock solution of 25 mg/mL, adding 50 μM of hemin slightly changes the pH of SD or CSM media. To eliminate the possible impact of an increased pH on filamentation, CSM media was adjusted to pH 5.8, the same pH as with additional hemin, while SD + hemin media was adjusted to pH 5.5, the same pH as SD. The 26 *C. albicans* isolates were cultured in CSM overnight at 30°C with shaking. Hyphal induction assays of 26 *C. albicans* isolates and biofilm formation assays of 10 isolates were conducted in CSM media with or without hemin (pH 5.8). Mutants were cultured in SD overnight at 30°C with shaking. Hyphal induction assays of mutants were assayed in SD with or without hemin (pH 5.5).

### Mutant construction

To manipulate the *C. albicans* genome, the transient CRISPR-Cas9 system was employed (Min *et al*. 2016). Briefly, the Cas9 cassette was amplified from the plasmid pV1093 (Vyas *et al*. 2015), and each of sgRNA cassette was generated by using split-joint PCR with “sgRNA/F YFG1” and “SNR52/R YFG1” as previously described in detail (Min *et al*. 2016; Huang *et al*. 2019). All primers and plasmids used in this are listed in Supplementary Table 1.

To construct *csa2*Δ/Δ in the SC5314 wild-type strain background, 2 halves of *CSA2* deletion cassettes were amplified from the plasmid pmh06 with primers “NAT1_CRIME/F” and “CSA2_NAT1/AR” and from the plasmid pmh05 with primers “CSA2_NAT1/AF” and “NAT1_CRIME/R,” respectively. SC5314 wild type was transformed with approximately 1 μg of Cas9 DNA cassette, 1 μg of CSA2-sgRNA DNA cassette, 2 μg of *NAT1_05*, and 2 μg of *NAT_06* repair template. Transformants were selected on YPD + NAT. Candidate colonies were further genotyped by PCR using primers “CAS2_CK/F” and “CAS2_CK_Int/R” for the absence of the *CAS2* ORF and using primers “CAS2_CK/F” and “NAT1_CK_Int/R” for the presence of the *NAT1* marker at the *CSA2* locus.

To construct *rbt5*Δ/Δ in the SC5314 strain background, the *RBT5* deletion cassette was amplified from the plasmid pSN69 with primers “RBT5_ARG4/AF” and “RBT5_ARG4/AR. MC79 strain was transformed with approximately 1 μg of Cas9 DNA cassette, 1 μg of RBT5-sgRNA DNA cassette, and 2 μg of *RBT5* deletion cassette. Transformants were selected on CSM media lacking arginine. Candidate colonies were further genotyped by PCR using primers “RBT5_CK/F” and “RBT5_CK_Int/R” for the absence of the *RBT5* ORF and using primers “RBT5_CK/F” and “ARG4_CK_Int/R” for the presence of the *ARG4* marker at the *RBT5* locus.

To construct *pga7*Δ/Δ in the SC5314 strain background, 2 halves of *PGA7* deletion cassettes were amplified from the plasmid pmh01 with primers “HIS1 CRIME/F” and “PGA7_HIS1/AR” and from the plasmid pmh02 with primers “PGA7_HIS1/AF” and “HIS1 CRIME/R,” respectively. Strain MC5 was transformed with approximately 1 μg of Cas9 DNA cassette, 1 μg of PGA7-sgRNA DNA cassette, 2 μg of *HIS1_01*, and 2 μg of *HIS1_02* repair template. Transformants were selected on CSM media lacking histidine. Candidate colonies were further genotyped by PCR using primers “PGA7_CK/F” and “PGA7_CK_Int/R” for the absence of the *PGA7* ORF and using primers “PGA7_CK/F” and “HIS1_CK_Int/R” for the presence of the *HIS1* marker at the *PGA7* locus.

To construct *pga10*Δ/Δ in the SC5314 strain background, 2 halves of *PGA10* deletion cassettes were amplified from the plasmid pmh01 with primers “HIS1 CRIME/F” and “PGA10_HIS1/AR” and from the plasmid pmh02 with primers “PGA10_HIS1/AF” and “HIS1 CRIME/R,” respectively. Strain MC5 was transformed with approximately 1 μg of Cas9 DNA cassette, 1 μg of PGA10-sgRNA DNA cassette, 2 μg of *HIS1_01*, and 2 μg of *HIS1_02* repair template. Transformants were selected on CSM media lacking histidine. Candidate colonies were further genotyped by PCR using primers “PGA10_CK/F” and “PGA10_CK_Int/R” for the absence of the *PGA10* ORF and using primers “PGA10_CK/F” and “HIS1_CK_Int/R” for the presence of the *HIS1* marker at the *PGA10* locus.

To construct the *csa2*Δ/Δ *rbt5*Δ/Δ double mutant in the SC5314 *rbt5*Δ/Δ strain background, 2 halves of *CSA2* deletion cassettes were prepared as described above for the *csa2*Δ/Δ mutant construction. The *rbt5*Δ/Δ strain was transformed with approximately 1 μg of Cas9 DNA cassette, 1 μg of CSA2-sgRNA DNA cassette, 2 μg of *NAT1_05*, and 2 μg of *NAT_06* repair template. Transformants were selected on YPD + NAT. Candidate colonies were further genotyped by PCR using primers “CAS2_CK/F” and “CAS2_CK_Int/R” for the absence of the *CAS2* ORF and using primers “CAS2_CK/F” and “NAT1_CK_Int/R” for the presence of the *NAT1* marker at the *CSA2* locus.

To construct the *pga7*Δ/Δ *pga10*Δ/Δ double mutant in the SC5314 *pga7*Δ/Δ strain background, 2 halves of *PGA10* deletion cassettes were amplified from the plasmid pmh06 with primers “NAT1_CRIME/F” and “PGA10_NAT1/AR” and from the plasmid pmh05 with primers “PGA10_NAT1/AF” and “NAT1_CRIME/R,” respectively. The *pga7*Δ/Δ strain was transformed with approximately 1 μg of Cas9 DNA cassette, 1 μg of CSA2-sgRNA DNA cassette, 2 μg of *NAT1_05*, and 2 μg of *NAT_06* repair template. Transformants were selected on YPD + NAT. Candidate colonies were further genotyped by PCR using primers “PGA10_CK/F” and “PGA10_CK_Int/R” for the absence of the *PGA10* ORF and using primers “PGA10_CK/F” and “NAT1_CK_Int/R” for the presence of the *NAT1* marker at the *PGA10* locus.

To construct *hap1*Δ/Δ (*zcf20*Δ/Δ) in the SC5314 strain background, 2 halves of *hap1* deletion cassettes were amplified from the plasmid pmh01 with primers “HIS1 CRIME/F” and “ZCF20_HIS1/AR” and from the plasmid pmh02 with primers “ZCF20_HIS1/AF” and “HIS1 CRIME/R,” respectively. Strain MC5 was transformed with approximately 1 μg of Cas9 DNA cassette, 1 μg of ZCF20-sgRNA DNA cassette, 2 μg of *HIS1_01*, and 2 μg of *HIS1_02* repair template. Transformants were selected on CSM media lacking histidine. Candidate colonies were further genotyped by PCR using primers “ZCF20_CK/F” and “ZCF20_CK_Int/R” for the absence of the *ZCF20* ORF and using primers “ZCF20_CK/F” and “HIS1_CK_Int/R” for the presence of the *HIS1* marker at the *ZCF20* locus.

To construct *hmx1*Δ/Δ in SC5314 strain background, 2 halves of *hmx1* deletion cassettes were amplified from the plasmid pmh01 with primers “HIS1 CRIME/F” and “HMX1_HIS1/AR” and from the plasmid pmh02 with primers “HMX1_HIS1/AF” and “HIS1 CRIME/R,” respectively. MC5 strain was transformed with approximately 1 μg of Cas9 DNA cassette, 1 μg of HMX1-sgRNA DNA cassette, 2 μg of *HIS1_01*, and 2 μg of *HIS1_02* repair template. Transformants were selected on CSM media lacking histidine. Candidate colonies were further genotyped by PCR using primers “HMX1_CK/F” and “HMX1_CK_Int/R” for the absence of the *HMX1* ORF and using primers “HMX1_CK/F” and “HIS1_CK_Int/R” for the presence of the *HIS1* marker at the *HMX1* locus.

### Hyphal induction assays and imaging

To assay hypha formation in *C. albicans* strains, cell culture and fixation were performed as described previously with minor modifications (Huang *et al*. 2019). Cells were harvested from overnight cultures, washed once with H_2_O, and then inoculated into indicated media at an OD_600_ of 0.5, following by 4-h incubation at 37°C with shaking. Cells were then collected and fixed with 4% formaldehyde for 15 min with vortexing. After washing twice with PBS, fixed cells were stained with Calcofluor-white (200 mg/mL) for 15 min. Stained cells were imaged using a Zeiss Axiovert 200 fluorescence microscope. Results were then quantified using the metric of percentage of hyphae, pseudohyphae, and yeast cells. Hyphal units and yeast cells were counted using multiple fields of view from a single assay to obtain the ratio of hyphal units to yeast cells. At least 100 cells were classified for each strain. Cells were classified as hyphae when the filaments were narrow (<2 μm) and had parallel sides. Cells were classified as pseudohyphae when the width was >2 μm, there was a constriction, there was a septum at the neck of the mother–daughter compartment, and the sides were not parallel. Cells were classified as yeast when a small bump protrudes from a parent cell or single round cells with the width of <5 μm. The cell length of the entire population was measured using ImageJ. At least 100 interseptal distance measurements were taken from 800 × 800 pixel fields of view. The workflow is as follows: Open the software → File → Open → Image file → Segmented Lane → Analyze → Label (Command T) → Analyze → Measure (Command M). The measured length in the unit of pixels was then normalized by a factor of 4.96 to the unit of micrometers.

Filamentation was assayed in 2 different ways. At issue is the fact that many of the non-SC5314 isolates make hyphae with shorter cell lengths than SC5314 and also make fewer hyphae than SC5314. Hence, all quantitative comparisons are imperfect. It is much less time consuming to count yeast, pseudohyphae, and hyphae than it is to measure cell lengths. Given that either approach is imperfect, we chose the less time-consuming approach. For experiments within the SC5314 background, such as the deletion mutant assays, it made sense to use the more time-consuming measure of cell length because it is more sensitive.

### Biofilm formation assay and imaging

Biofilm production and imaging followed previous published methods with minor modifications (Lanni *et al*. 2020; Do *et al*. 2022; Cravener *et al*. 2023). To assay biofilm formation in 96-well plates, cells were grown in 5 mL of CSM for overnight at 30°C, and then cells were washed with H_2_O and transferred to 100 μL of prewarmed CSM (pH 5.8) and CSM supplemented with 50 μM of hemin (pH 5.8) to an OD_600_ of 0.05, in wells of a 96-well plate (Greiner, 655090). The cells were incubated in a shaker incubator at 37°C for 90 min with mild shaking (60 rpm) to allow for adherence to the bottom of a 96-well plate, and then each well was gently washed twice with PBS to remove nonadhered cells. One hundred microliters of prewarmed assay media was added into each well, and cells were allowed to form biofilm in a shaker incubator with 60 rpm at 37°C for 24 h. In the next day, media was carefully discarded from each well, and biofilms were fixed by incubation with 100 μL of 4% formaldehyde in PBS solution for 1 h at room temperature (RT) and then gently washed twice with PBS. Fixed biofilms were stained with Calcofluor-white (200 μg/mL in PBS) overnight at RT with mild shaking (60 rpm), and then each well was gently washed twice with PBS. For clarification and refractive index matching, biofilms were incubated with 50% of 2,2′-thiodiethanol (TDE) in PBS for 1 h at RT, and then 100% TDE was added to each biofilm. Biofilms were imaged by using a Keyence fluorescence microscope with a Keyence 20× objective and 2× zoom.

### RNA extraction and RNA sequencing

RNA extraction was performed as previously described (Cravener and Mitchell 2020). To prepare the RNA, cells were grown in 5 mL of SD (pH 5.5) overnight at 30°C. The next day, cells were harvested, washed, and then inoculated with an initial OD_600_ of 0.02 in 30 mL of media, including SD (pH 5.5), SD added with 50 mM hemin (pH 5.5), and SD (buffered with 50 mM HEPES, pH 7.0) as indicated. After 4 h at 37°C, cells of 25 mL were harvested by vacuum filtration and quickly frozen at −80°C until RNA extraction. Cells of the remaining 5 mL were harvested and treated the same as filamentation assay for imaging. Three cultures of each strain were grown to provide 3 biological replicates for mRNA-level detection. Cell disruption was achieved mechanically using Zirconia beads (Ambion, Fisher Scientific, Waltham), and RNA extraction was performed using a 25:24:1 phenol:chloroform:isoamyl alcohol method combined with a Qiagen RNeasy Mini Kit (Qiagen, catalog number 74104).

A total amount of 1 μg RNA per sample was used as input material for RNA-seq. Sequencing libraries were generated by purifying messenger RNA from total RNA using poly-T oligo–attached magnetic beads. The first cDNA was synthesized using random hexamer primers, followed by sequencing of both ends of cDNA fragments using the Illumina platform (Novogene). Sequencing reads were aligned to *C. albicans* reference (Assembly 22) using Hisat2 v2.0.5. Differential expression analysis between 2 groups (3 biological replicates per group) was performed using the DESeq2 v1.40.2 R package using alpha = 0.05 (Love *et al*. 2014).

### Software

Images were compiled, and any adjustments were performed in ImageJ (Schindelin *et al*. 2012). Single guide RNA sequences were checked for specificity using Cas-OFFinder software (Bae *et al*. 2014). Analyses were performed with GraphPad Prism version 8.00 (GraphPad Software, Inc., La Jolla). Venn diagrams were constructed by Venn Diagrams software (http://bioinformatics.psb.ugent.be/webtools/Venn/). Genes for the Venn diagrams were defined as follows: Blankenship: columns with liquid media (10% FBS + YPD, Lee's media, RPMI-MOPS media, or spider media at 37°C vs YPD at 30°C) where genes had a fold change of greater than 2 and were statistically significant (176 genes); Cravener: published hypha-associated gene list (152 genes); SD + hemin vs SD (pH 5.5): genes with a fold change of greater than 2 and a *P*adj < 0.05 (139 genes); and SD (pH 7.0) vs SD (pH 5.5): genes with a fold change of greater than 2 and a *P*adj < 0.05 (245 genes). To perform Gene Ontology (GO) term analyses, we implemented clusterProfiler (v4.8.1) in R by creating a GO term library using FungiDB (Candida albicans.Eupath.v63) with the R AnnotationForge package (Wu *et al*. 2021). Genes were defined by having an adjusted *P* < 0.05 and a fold change on a log_2_ scale of > 1. Only GO categories with a *P* < 0.05 were considered significant.

## Results and discussion

### Natural variation in hemin-induced filamentation

**Fig. 1.**
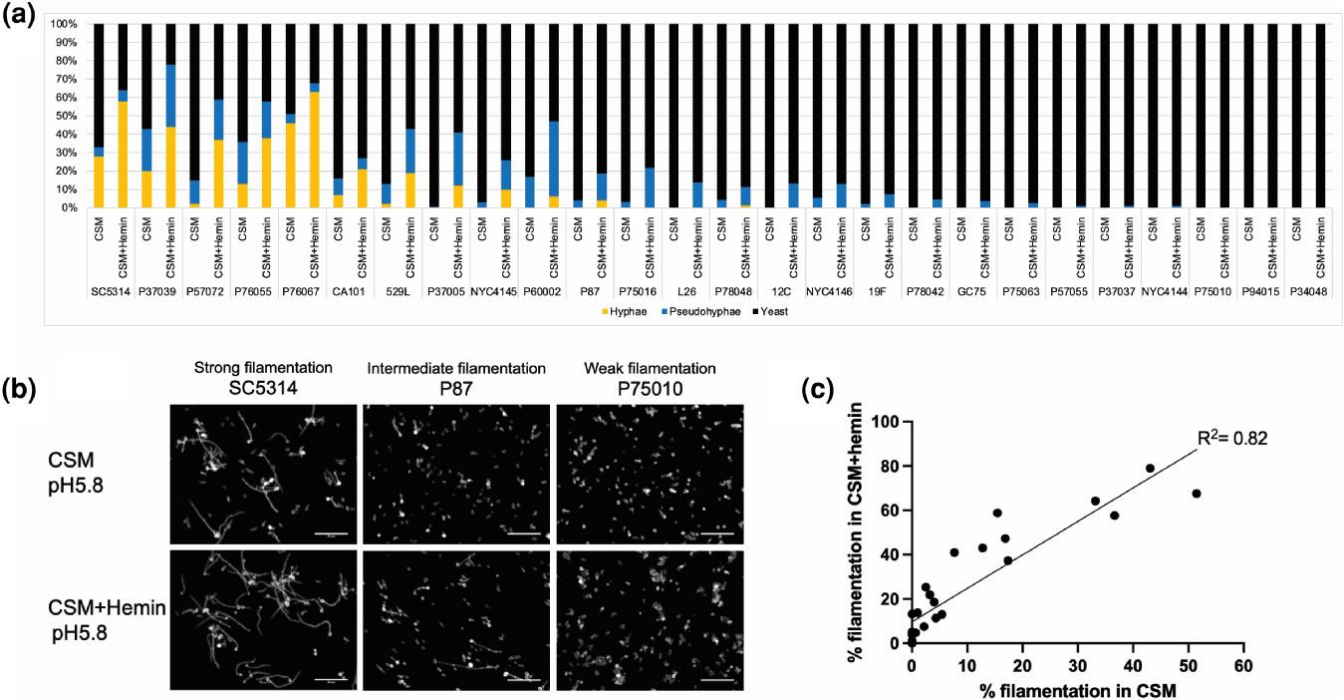
Hemin-induced filamentation among *C. albicans* isolates. a) Quantification of filamentation. Planktonic filamentation of 26 *C. albicans* isolates was assayed in CSM and CSM + hemin at pH 5.8 at 37°C for 4 h. Cell types were counted and are presented as ratios in indicated background. Quantitative analysis used multiple fields of view from a single assay. Yellow bars indicate hyphae, blue bars indicate pseudohyphae, and black bars indicate yeast. b) Representative fields. *Candida albicans* clinical isolates SC5314 (strong filamentation), P87 (intermediate filamentation), and P75010 (weak filamentation) are shown in CSM and CSM + hemin (pH 5.8). The white scale bar in this panel is 50 μm in length. c) Correlation analysis of filamentation. The level of filamentous cells (total percentage of hyphae and pseudohyphae) in CSM vs CSM + hemin is graphed for the 26 isolates. The correlation is represented by an *R*^2^ = 0.82.

We sought to survey species-level variation in the hemin-induced filamentation response of *C. albicans*. To that end, we assayed 23 clinical isolates and 3 oak tree isolates for filamentation in CSM media with or without 50 μM hemin at 37°C (Supplementary Fig. 1). For 17 strains, the CSM control samples had some hyphal and pseudohyphal cells, comprising up to ∼50% of the population (Fig. 1a and b). These filamentous cells were apparently induced by the shift to 37°C temperature alone. In CSM + hemin, the 17 strains had a higher level of hyphal and pseudohyphal cells than in CSM, and an additional 6 strains produced some pseudohyphal cells (Fig. 1a). The level of filamentous cells in the presence of hemin correlated well with the level of filamentous cells in the absence of hemin (Fig. 1c). These results suggest that strain variation in hemin-induced filamentation reflects variation in overall filamentation ability.

### Hemin-induced biofilm formation

**Fig. 2.**
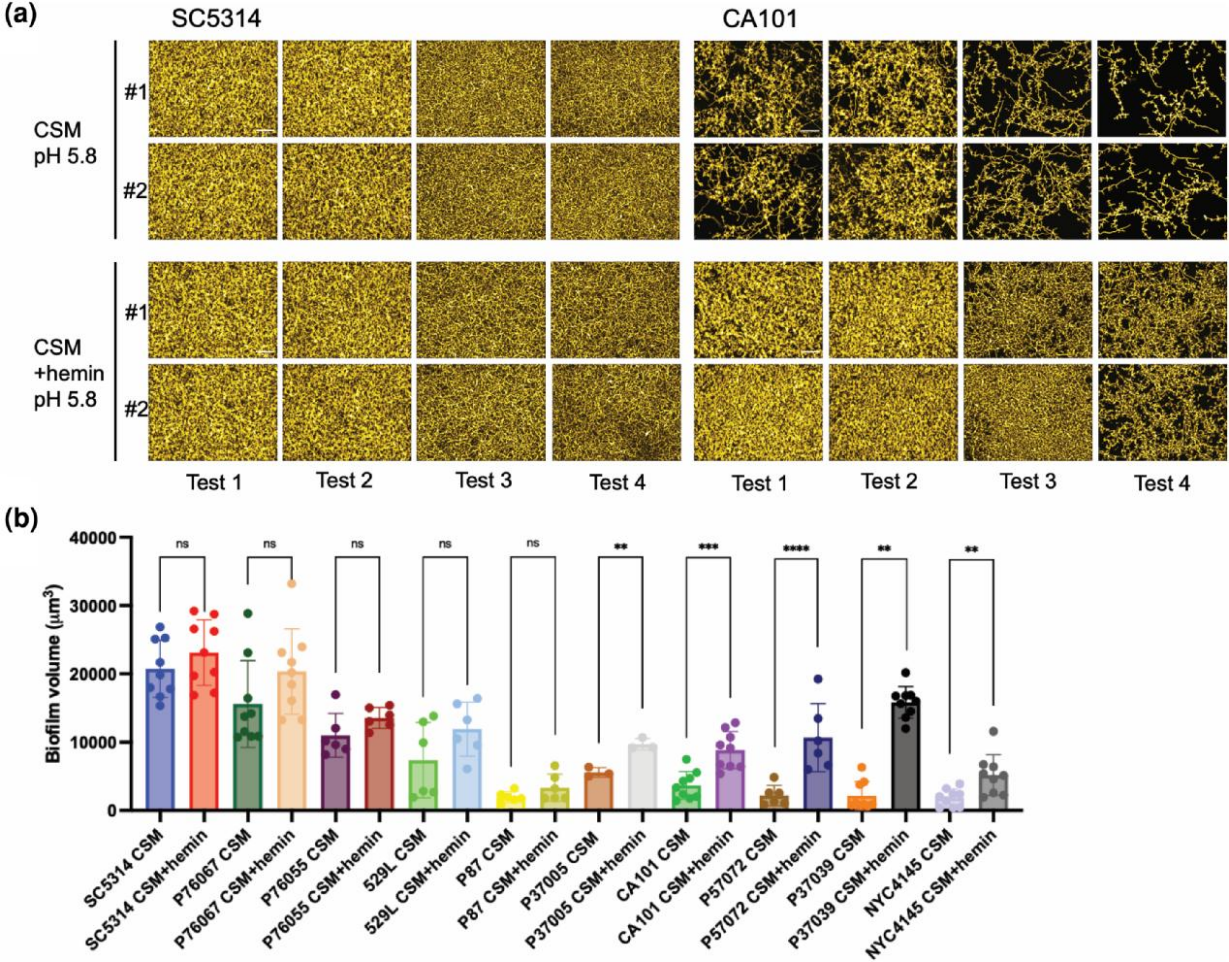
Hemin-induced biofilm formation. a) Biofilm apical view projections. Four replicate biofilm assays are shown for *C. albicans* isolates SC5314 and CA101. Strains were grown in 96-well plates in CSM and CSM + hemin at 37°C for 24 h. Fixed biofilms were stained with Calcofluor-white, clarified with TDE, and then imaged with a Keyence fluorescence microscope. Representative apical views are shown. White scale bars in each panel are 50 μm in length. b) Biofilm volume quantification analysis. Ten *C. albicans* isolates were assayed for biofilm formation as described for panel. Biofilm formation was assayed with 3 replicates in each assay and up to 4 independent assays. a) Biofilm volume was measured with ImageJ. Each dot indicates 1 biologically independent sample. *t*-tests indicated significant differences between CSM and CSM + hemin for strains P37005, CA101, P57072, P37039, and NYC4145 strains. ns, not significant, **P < 0.01, ***P < 0.001, and ****P < 0.0001.

Many growth conditions that promote hypha formation also promote biofilm formation. To determine whether hemin may be such a signal, we examined 10 *C. albicans* isolates for biofilm formation in CSM media with or without hemin. Effects were quantified with multiple replicates (Fig. 2a), using measurement of biofilm volume from image stacks (Fig. 2b). Five strains produced comparable levels of biofilm in the presence and absence of hemin (Fig. 2b). This group included the wild-type strain SC5314 and others that were strong biofilm formers in the absence of hemin. Five strains produced higher levels of biofilm in the presence of hemin than in its absence (Fig. 2b). This group included the nonpathogenic strain CA101 (Schonherr *et al*. 2017) and other strains that produced biofilm weakly in the absence of hemin. These results indicate that hemin can stimulate biofilm formation, with effects most evident among weaker biofilm formers under our conditions. A simple explanation for most of the responsive strains is that hemin-stimulated hypha formation improves adherence, increasing the adherent population size and thus increasing overall biofilm production. Gene expression data below support this explanation; hemin causes upregulation of adhesin genes that include *ALS3* and *HWP1* and causes downregulation of the antiadhesin gene *YWP1*.

### Hemin-induced gene expression

To investigate the transcriptional response of *C*. *albicans* to hemin, global gene expression was assayed by RNA-seq. We used SD media at pH 5.5 for these studies because it supports pronounced hyphal induction by hemin for the wild-type reference strain SC5314 (Supplementary Fig. 2). (We had used CSM and CSM + hemin media in our multistrain surveys above because many strains did not undergo filamentation or produce biofilm in SD or SD + hemin media.) We used SD media buffered to pH 7.0 as a different hypha-inducing condition (Supplementary Fig. 2) for comparison, because the response to neutral/alkaline pH has been characterized extensively (Davis 2009). RNA samples were extracted from *C. albicans* SC5314 cells grown for 4 h at 37°C in SD (pH 5.5), SD + hemin (pH 5.5), and SD (pH 7.0).

**Fig. 3.**
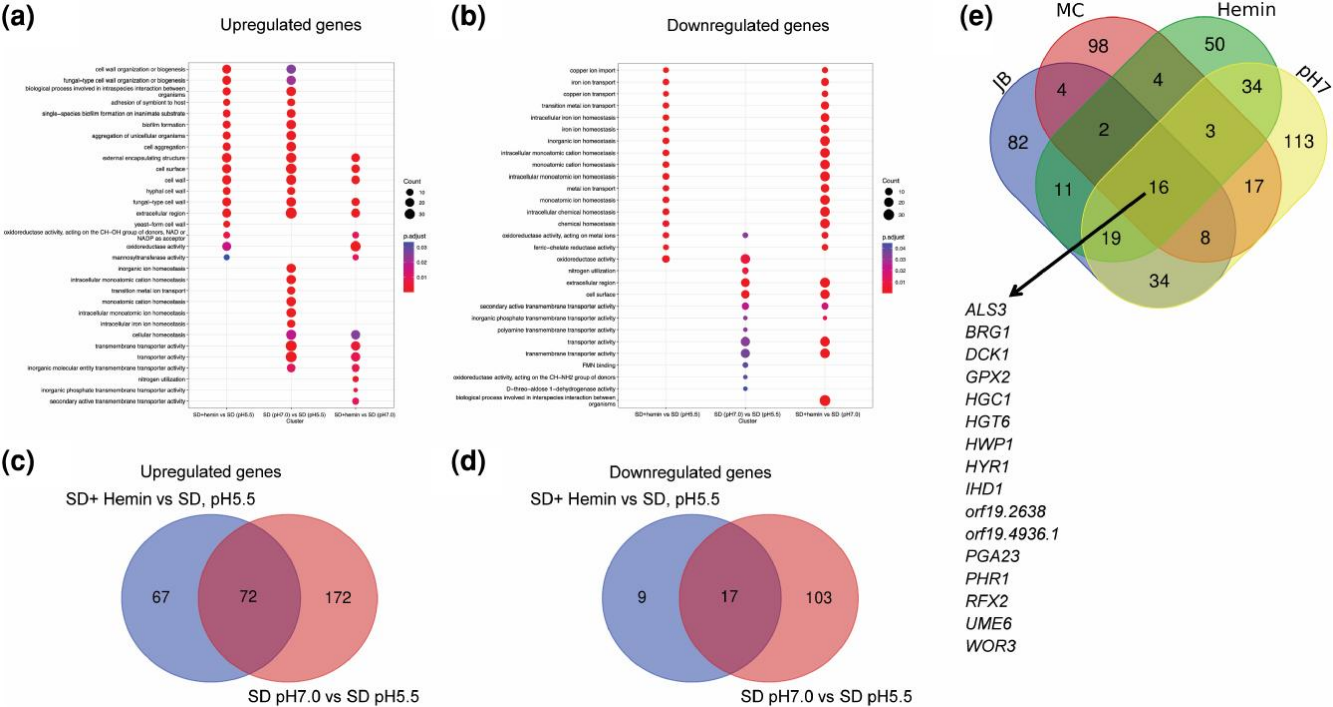
Hemin-induced gene expression. a, b) GO enrichment analysis using clusterProfiler (Wu *et al*. 2021) for genes upregulated or downregulated with >2-fold changes and *P* < 0.05 in SD + hemin vs SD (pH 5.5), buffered SD (pH 7.0) vs SD (pH 5.5), and SD + hemin vs buffered SD (pH 7.0), respectively. The dot size represents the number of genes in each category, and categories with a *P* < 0.05 were considered significant. c, d) Venn diagrams depict the genes upregulated c) or downregulated d) with a 2-fold change and a *P* < 0.05 SD + hemin vs SD (pH 5.5) and SD (pH 7.0) vs SD (pH 5.5). e) Venn diagram of hemin-induced genes, neutral pH-induced genes, and Blankenship “JB” (Azadmanesh *et al*. 2017) and Cravener “MC” (Cravener *et al*. 2023) hypha-associated gene sets. The 16 common hypha-associated genes are listed.

We compared SD + hemin (pH 5.5) vs SD (pH 5.5) to define hemin-responsive gene expression changes (log fold change >1 or <−1 and *P*adj < 0.05). We identified 172 upregulated genes and 27 downregulated genes (Supplementary Table 2). Upregulated genes included many hypha-associated genes (*HWP1*, *ECE1*, *HYR1*, *HGC1*, *ALS3*, etc.) and were enriched for GO terms related to cell wall, adherence, and biofilm formation (Fig. 3a). The heme oxygenase gene *HMX1* and its transcriptional activator gene *HAP1* were also upregulated, though many other Hap1 targets (Andrawes *et al*. 2022) were not. Downregulated genes included the yeast-associated gene *YWP1* and iron transport genes (*CFL4*, *FRE10*, *FTR1*, etc.) and were enriched for GO terms related to metal ion homeostasis (Fig. 3b). Downregulation of iron transport genes likely reflects the excess iron provided by hemin supplementation. The overall response to hemin makes sense in view of hemin as an inducer of hyphae and biofilm as well as an iron source.

We compared SD (pH 7.0) vs SD (pH 5.5) to define pH-responsive gene expression changes (log fold change >1 or <−1 and *P*adj < 0.05). We identified 290 upregulated genes and 132 downregulated genes (Supplementary Table 2). Upregulated genes included many hypha-associated genes as well as alkaline pH-induced genes (*GAP1*, *ENA2*, *PHO89*, etc.; Bensen *et al*. 2004) and were enriched for GO terms related to cell wall and adherence as well as ion homeostasis (Fig. 3a). Downregulated genes included *YWP1* as well as alkaline pH-repressed genes (*PHR2*, *CRZ2*, *RIM8*, etc.; Bensen *et al*. 2004) and were enriched for GO terms related to the cell surface and transmembrane transport (Fig. 3b). The overall response to this pH change is consistent with previous studies and provides the added resolution of RNA-seq compared to microarray detection.

Each of the 2 comparisons above presents gene expression changes associated with induction of hypha formation. Genes that are upregulated in both comparisons included many with roles in hypha or biofilm formation. Two recent studies define hypha-associated genes through a consensus among diverse RNA-seq samples. Cravener *et al*. (2023) identified 152 genes with expression levels that correlate with hypha formation ability among 17 strains grown in RPMI + serum at 37°C. The Blankenship group (Azadmanesh *et al*. 2017) identified genes that were upregulated under diverse hypha-inducing conditions in both liquid and solid media. (For analysis here, we considered only the 176 genes identified from their liquid media samples.) Overlap between those 2 consensus sets of hypha-associated genes was limited to 30 genes. Among those 30 genes, 16 were also upregulated in both of our comparisons: *ALS3*, *BRG1*, *DCK1*, *GPX2*, *HGC1*, *HGT6*, *HWP1*, *HYR1*, *IHD1*, *orf19.2638*, *orf19.4936.1*, *PGA23*, *PHR1*, *RFX2*, *UME6*, and *WOR3*. Overlap with both the Blankenship and Cravener hypha-associated gene sets was better with pH 7-induced genes than with hemin-induced genes, which may reflect the use of neutral/alkaline growth conditions in both consensus studies (Fig. 3e).

### Genetic control of hemin-induced filamentation

**Fig. 4.**
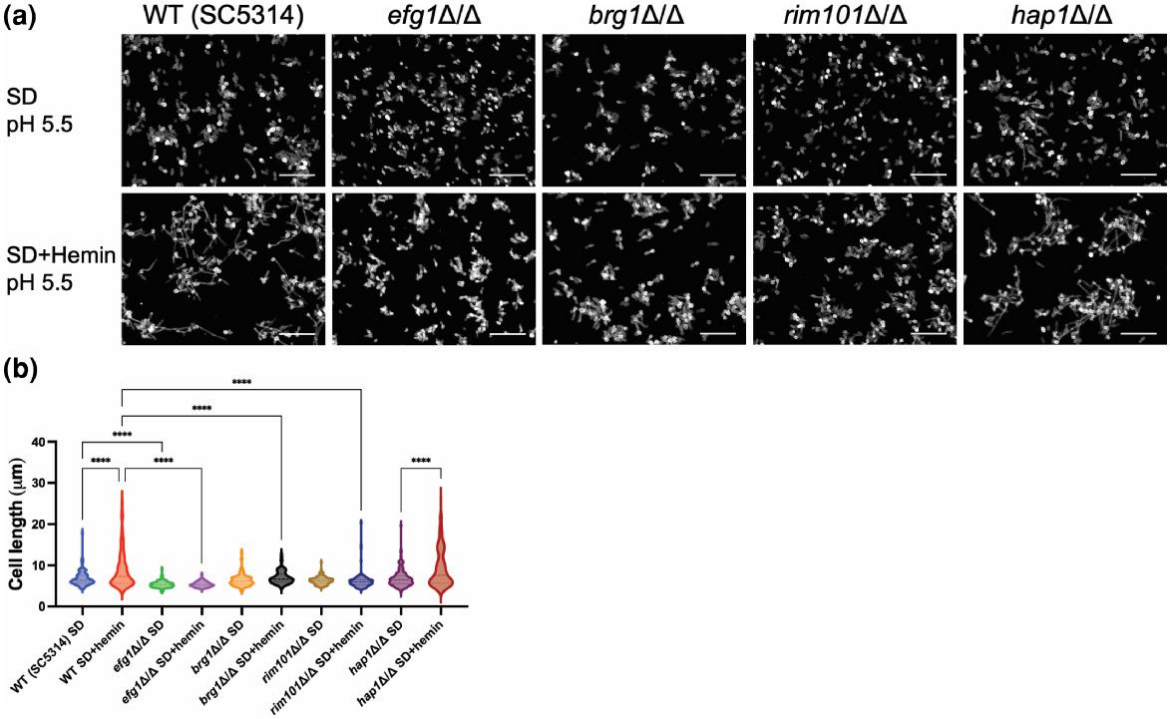
Hemin-induced filamentation in mutant strains. a) Planktonic filamentation of wild-type SC5314, *efg1*Δ/Δ, *brg1*Δ/Δ, *rim101*Δ/Δ, and *hap1*Δ/Δ were examined in SD (pH 5.5) and SD + hemin (pH 5.5) at 37°C for 4 h. White scale bars indicate 50 μm in length. b) Boxplots of the overall cell body lengths measured from the indicated clinical isolate background. Quantification was performed with a single microscopic field. Significant differences in mean cell unit length between isolates are indicated (1-way ANOVA, ****P < 0.0001). The *efg1*Δ/Δ, *brg1*Δ/Δ, and *rim101*Δ/Δ strains had nonsignificant differences between SD and SD + hemin.

To begin to define regulators that may mediate hemin-induced filamentation, we assayed filamentation in SD media with or without hemin in select mutant strains. We included *efg1*Δ/Δ and *brg1*Δ/Δ mutants, defective in 2 master regulators of hypha and biofilm formation; a *rim101*Δ/Δ mutant, defective in a pH response regulator that is required for growth on hemoglobin; and *hap1*Δ/Δ, defective in a regulator of heme–iron acquisition (Andrawes *et al*. 2022). Filamentation was significantly induced by hemin in the wild-type strain SC5314, but not in the *efg1*Δ/Δ or *brg1*Δ/Δ mutants (Fig. 4a and b). This result indicates that the hemin response depends upon known hypha/biofilm master regulators. Filamentation was not significantly induced by hemin in the *rim101*Δ/Δ mutant (Fig. 4a and b). This result indicates that Rim101 is required both for pH-induced filamentation (Davis 2009) and hemin-induced filamentation. Filamentation was significantly induced by hemin in the *hap1*Δ/Δ mutant (Fig. 4a and b). In addition, hemin induced filamentation in mutants defective in the heme oxygenase Hmx1 and in the surface heme-binding proteins Rbt5, Pga10, Pga7, and Csa2 (Supplementary Fig. 3), all of which depend upon Hap1 for expression (Andrawes *et al*. 2022). These results suggest that heme–iron acquisition is not required for hemin-induced filamentation.

How does hemin induce filamentation? Given that Hap1 and its major target genes are not required for hemin-induced filamentation, we suggest that hemin may interact with a surface receptor to effect a morphogenic response. Many surface- and membrane-associated proteins have roles in filamentation (Chen *et al*. 2020). Given that the pH response regulator Rim101 is required for hemin-induced filamentation, the upstream pH response pathway components would be good candidates. Rim101 activity is regulated by the plasma membrane proteins Rim21 and Dfg16 (Davis 2009); hence, both gene products are candidate hemin receptors. In fact, both *RIM21* and *DFG16* are required for competitive growth on heme, based on transposon mutagenesis (Andrawes *et al*. 2022). It will be exciting to see whether these genes govern hemin-induced filamentation as well.

### Conclusion

- Among multiple clinical and environmental *C. albicans* isolates, the filamentation response to hemin varies in proportion to its filamentation level in the absence of hemin.
- Hemin can induce biofilm formation in a subset of *C. albicans* isolates.
- A set of 72 genes is upregulated in response to filamentation induced by either hemin or neutral pH. The gene expression response to hemin seems to reflect both its ability to induce filamentation and its ability to provide iron.
- Hemin-induced filamentation response depends upon filamentation regulators Efg1, Brg1, and Rim101, but not upon heme acquisition regulator Hap1 or several heme-associated gene products under Hap1 control.

## Supplementary Material

jkae053_Supplementary_Data

## Data Availability

Strains and plasmids are available upon request. The authors affirm that all data necessary for confirming the conclusions of the article are present within the article, figures, and tables. RNA-seq data are available through the NCBI under BioProject ID PRJNA1036256. Supplemental material available at G3 online.
