# Supplementary material for: Regulatory features of Candida albicans hemin-induced filamentation
Source: G3 (Bethesda). 2024 Mar 12;14(5):jkae053. doi: 10.1093/g3journal/jkae053 (PMC11075532; doi:10.1093/g3journal/jkae053)
Supplement: jkae053_Supplementary_Data [file jkae053_supplementary_data.zip › Figure_S1_G3-2024-404912.pdf]

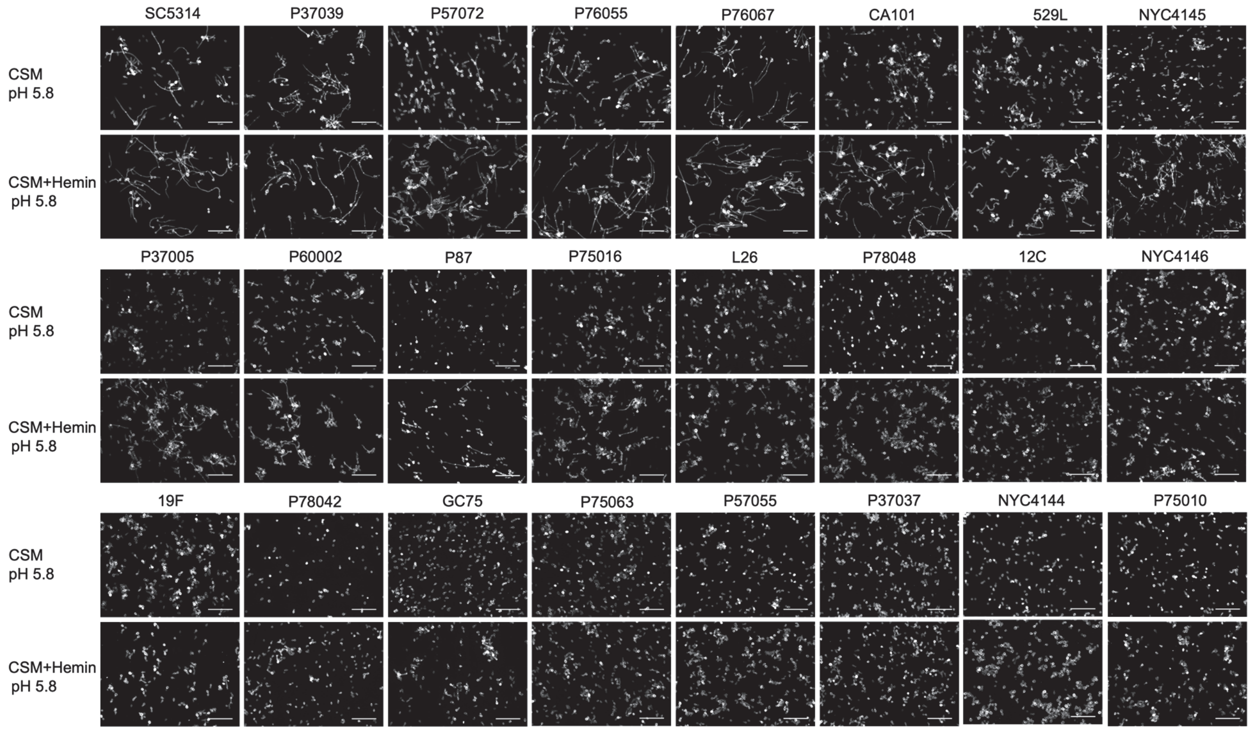

Figure S1. Cell morphology of 24 *C. albicans* isolates grown in CSM (pH 5.8) and CSM +hemin (pH 5.8) media at 37°C for 4 hours. The white scale bar indicates 50 μm in length. Top panel includes SC5314, P37039, P57072, P76055, P76067, CA101, 529L, and NYC4145. Middle panel includes P60002, P37005, P87, P75016, L26, P78048, NYC4146, and 19F. Bottom panel includes 12C, P73037, P78042, P57055, GC75, P75063, NYC4144, P75010. Images of P94015 and P34048 are not shown.
