# Supplementary material for: Regulatory features of Candida albicans hemin-induced filamentation
Source: G3 (Bethesda). 2024 Mar 12;14(5):jkae053. doi: 10.1093/g3journal/jkae053 (PMC11075532; doi:10.1093/g3journal/jkae053)
Supplement: jkae053_Supplementary_Data [file jkae053_supplementary_data.zip › Figure_S2_G3-2024-404912.pdf]

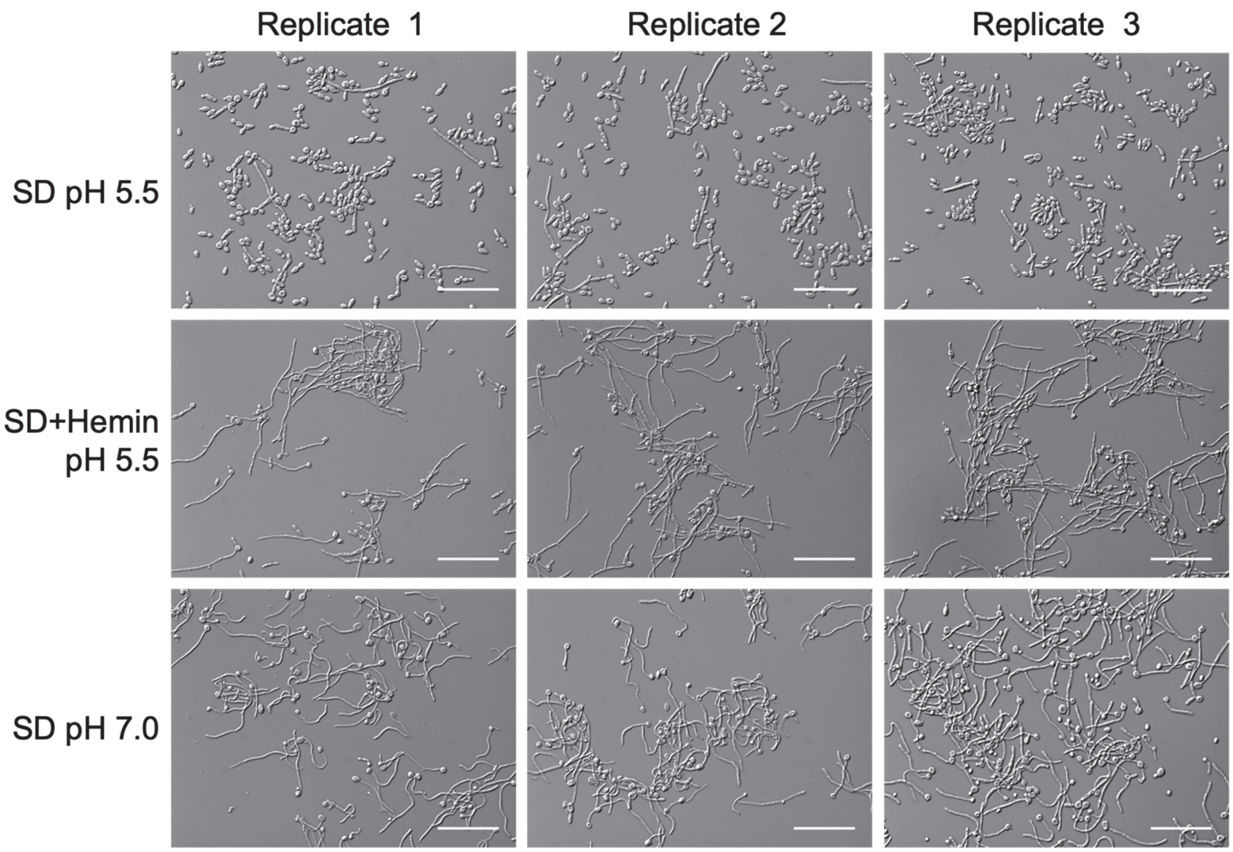

Figure S2. Cell morphology of cultures used for RNA samples. *C. albicans* SC5314 cells were grown in SD (pH 5.5), SD+hemin (pH 5.5), and SD (pH 7.0) at 37°C for 4 hours in three biological replicates. White scale bars indicate 50 µm in length.
