# Supplementary material for: Regulatory features of Candida albicans hemin-induced filamentation
Source: G3 (Bethesda). 2024 Mar 12;14(5):jkae053. doi: 10.1093/g3journal/jkae053 (PMC11075532; doi:10.1093/g3journal/jkae053)
Supplement: jkae053_Supplementary_Data [file jkae053_supplementary_data.zip › Figure_S3_G3-2024-404912.pdf]

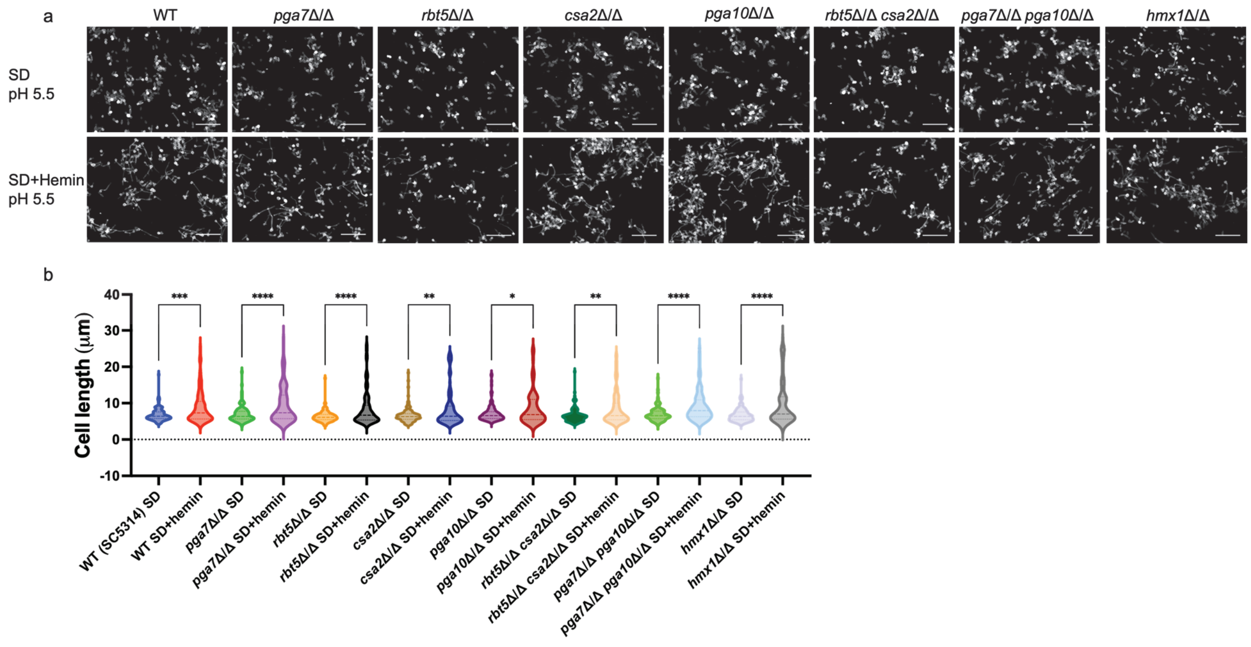

Figure S3. (a) Cell morphology of *C. albicans* SC5314 and its gene deletion mutants that were grown in SD and SD+hemin (pH 5.5) at 37°C for 4 hours. White scale bars indicate 50 μm in length. Mutants include *csa2*Δ/Δ, *rbt5*Δ/Δ, *pga7*Δ/Δ, *pga10*Δ/Δ, *csa2*Δ/Δ*rbt5*Δ/Δ, *pga7*Δ/Δ*pga10*Δ/Δ, and *hmx1*Δ/Δ. (b) Boxplots of the overall cell body lengths measured from the indicated clinical isolate background. Quantification was performed with a single microscopic field. Significant differences in cell length of each strain between SD and SD+hemin are indicated (one-way ANOVA, * P<0.05, ** P<0.01, *** P<0.001, and **** P<0.0001). The wild type and its mutants had non-significant differences in SD and in SD+hemin.
