# Supplementary material for: Regulatory features of Candida albicans hemin-induced filamentation
Source: G3 (Bethesda). 2024 Mar 12;14(5):jkae053. doi: 10.1093/g3journal/jkae053 (PMC11075532; doi:10.1093/g3journal/jkae053)
Supplement: jkae053_Supplementary_Data [file jkae053_supplementary_data.zip › Supplemental_Material_Legends_G3-2024-404912.docx]

**Figure S1.** Cell morphology of 24 *C. albicans* isolates grown in CSM (pH 5.8) and CSM +hemin (pH 5.8) media at 37°C for 4 hours. The white scale bar indicates 50 μm in length. Top panel includes SC5314, P37039, P57072, P76055, P76067, CA101, 529L, and NYC4145. Middle panel includes P60002, P37005, P87, P75016, L26, P78048, NYC4146, and 19F. Bottom panel includes 12C, P73037, P78042, P57055, GC75, P75063, NYC4144, P75010. Images of P94015 and P34048 are not shown.

**Figure S2.** Cell morphology of cultures used for RNA samples. *C. albicans* SC5314 cells were grown in SD (pH 5.5), SD+hemin (pH 5.5), and SD (pH 7.0) at 37°C for 4 hours in three biological replicates. White scale bars indicate 50 μm in length.

**Figure S3.** (a) Cell morphology of *C. albicans* SC5314 and its gene deletion mutants that were grown in SD and SD+hemin (pH 5.5) at 37°C for 4 hours. White scale bars indicate 50 μm in length. Mutants include *csa2*Δ/Δ, *rbt5*Δ/Δ, *pga7*Δ/Δ, *pga10*Δ/Δ, *csa2*Δ/Δ*rbt5*Δ/Δ, *pga7*Δ/Δ*pga10*Δ/Δ, and *hmx1*Δ/Δ. (b) Boxplots of the overall cell body lengths measured from the indicated clinical isolate background. Quantification was performed with a single microscopic field. Significant difference in cell length of each strain between SD and SD+hemin are indicated (one-way ANOVA, * P<0.05, ** P<0.01, *** P<0.001, and **** P<0.0001). The wild type and its mutants had non-significant differences in SD and in SD+hemin.

**Table S1.** Strains, primers, and plasmids used in this study.

**Table S2.** RNA-seq data set for SD+hemin vs SD (pH 5.5), buffered SD (pH 7.0) vs SD (pH 5.5), SD+hemin vs buffered SD (pH 7.0).
